# Laparoscopic myomectomy

**Published:** 2014

**Authors:** RA Stoica, I Bistriceanu, R Sima, N Iordache

**Affiliations:** * General Surgery Department, “Sf. Ioan” Clinical Emergency Hospital, Bucharest, Romania; ** Endocrinology Department, Emergency Hospital Craiova, Craiova, Romania; *** ”Bucur” Clinic of Obstetrics and Gynecology, Bucharest, Romania

**Keywords:** laparoscopic myomectomy, fibroid, adhesion, infertility

## Abstract

Uterine leiomyoma is the most common benign tumour occurring in women in the reproductive age. It is typically found during the middle and later reproductive years. The prevalence quoted in literature ranges from 20-50% based on post mortem studies. The symptoms usually reported by women with fibroids are the following: abnormal gynaecologic haemorrhage, chronic pelvic pain, dyspareunia, as well as urinary and bowel symptoms, urinary frequency or retention and, in some cases, infertility. During pregnancy, premature labor might be caused, interfering with the position of the fetus or abortion could be induced. However, only 30% of the women develop symptoms, most of them being asymptomatic.

It was proved that the factors that can cause fibroids are the following: genetic, hormonal, and growth factors, especially transforming the growth factor beta (TGFb)-related cellular changes.

As diagnosis tools, studies are revealing that ultrasound has been shown to be an insufficient method of myoma mapping, and magnetic resonance imaging should be preferred for surgical therapy planning. The contour of the endometrial cavity is delineated by using trans vaginal ultrasound and saline infusion hysterosonography, but hysteroscopy is the gold standard to evaluate the uterine cavity.

## Introduction

Uterine leiomyoma is the most common benign tumour occurring in women in the reproductive age. It is typically found during the middle and later reproductive years. The prevalence quoted in literature ranges from 20-50% based on post mortem studies [**1**]. However, only 30% of women develop symptoms [**2**], most of them being asymptomatic [**3**].

It was proved that the factors that could cause fibroids are the following: genetic, hormonal, and growth factors, especially transforming the growth factor beta (TGFb)-related cellular changes [**4**].

As diagnosis tools, studies are revealing that ultrasound has been shown to be an insufficient method of myoma mapping, and magnetic resonance imaging should be preferred for surgical therapy planning [**5**]. The contour of the endometrial cavity is delineated by using trans vaginal ultrasound and saline infusion hysterosonography, but hysteroscopy is the gold standard to evaluate the uterine cavity [**6**].

**Laparoscopy or laparotomy?**

Laparoscopic myomectomy was first described in 1979 (for subserosal fibroids) [**7**] and in the early '90s, the procedure started to be used also for intramural myomas also [**8**,**9**]. Nowadays, laparoscopic myomectomy is one of the common surgical procedures in infertile patients.

Indications: the presence of a subserosal or intramural fibroid that narrows the uterine cavity, myomas greater than 3cm and multiple fibroids. The feasibility of laparoscopic myomectomy has already been shown in numerous clinical studies. A consensus that the maximal size must be of 8-10cm gradually emerged and the total number of fibroids should not exceed four [**10**].

As a major risk after myomectomy, what should be mentioned is the uterine rupture during pregnancy or during labor. Factors that lead to this are the following: excessive coagulation, uterine fistulas, hematomas intramurally formed, and the wrong suture size. As far as laparotomies the multilayer and uterine suturing are allowed if they are optimally closed after enucleating. Laparoscopic surgeons are trying to adopt multilayer techniques instead of single layer in order to prevent uterine rupture [**11**].

Other complications that could appear with the laparoscopic myomectomy are the following: embolism, thrombosis, bowel injury, ureteral injuring, urinary bladder injury, excessive bleeding, and fistula.

Laparoscopic myomectomy has advantages compared with laparotomy. Women reported a lower intensity of post op pain, shorter recovery time, shorter hospital stay, less ileus time. Dubuisson et al., threw the second-look laparoscopy and discovered an adhesion rate of 35,6% after laparoscopic myomectomy and 90% following laparotomy [**12**]. The incidence is the highest in the posterior uterine incisions.

**Fibroids and infertility**

Uterine fibroids are detected in a small but significant number of infertile women. However, the impact of leiomyoma on infertility is controversial.

Fibroids are present in approximately 5-10% of the patients presenting with infertility; however, they were found to be the sole identified factor in only 1-2,4% of the infertile patients [**13**,**14**].

Andreoli researched the statistics of many authors and concluded that out of 3712 cases of myomectomies for fibroids associated with infertility, 1311 patients (36,1%) got pregnant [**15**]. 

In their 10 years of experience, Buttram and Reiter, found uterine fibroids to be the sole cause of infertility in only 2,4% of the cases [**16**]. Panait Sirbu et al. found the fibroids as a sole cause of infertility in 5,31% of cases [**15**].

The mechanisms by which myomas can lead to infertility are complex. The uterine contractility is disturbed and this fact interferes with sperm and ovum transport [**16**-**18**]. The tubal ostia could be obstructed by the fibroid. Adhesions around salpinges could result from subserosal myomas [**15**]. The distortion and enlargement of the endometrial cavity by submucous and intramural fibroids with an intracavitary component can affect the implantation [**19**,**20**]. Implantation is also affected by vascular disturbances and inflammation of the endometrium and secretion of vasoactive substances [**16**,**21**]. Submucosal fibroids are associated with a 70% reduction in the delivery rate and intramural fibroids with a 30% rate [**22**].

If a causal relationship between myomas and infertility can be established, treatment is indicated in order to enhance fertility.

A comparative study in literature that researched the pregnancy rate in women who have had laparoscopic myomectomy and a control group that did not have any surgery, was found [**23**]. Infertile women who did not have any other cause of infertility were included in the study. 106 were operated women and 106 did not receive treatment, were followed for 9 months. The pregnancy rate was 42% in the surgical group versus 11%.

From a study of 1941 patients who have had laparoscopic myomectomy, it could be seen the abortion rate improved from 41% before surgery to 19% after myomectomy [**16**].

Of course, there is always the risk of recurrence. This risk was estimated to be 11.7 and 36.1 after one and 3 years following the laparoscopic myomectomy (multicenter study) [**24**].

Laparoscopic myomectomy was performed on 17 women who were diagnosed with uterine fibroids, with ages between 20 and 45 years old (average age was 30 years old) since January 1st 2011 until January 1st 2013. The most frequent symptoms were the following: abnormal gynaecologic haemorrhage, pelvic pains, dyspareunia, infertility (14 patients). One of the patients (20 years old) did not become sexually active by that age and 2 of them did not wish to get pregnant in the future, so they asked to also perform sterilization by ligaturing the salpinges. The sterilization was performed. However, they did not agree with hysterectomy for personal reasons.

10 out of 17 patients had intramural fibroids and the rest had subserosal ones. Surgery was not performed in any case of inwards leiomyomas. The fibroids' sizes were between 2 and 10cm and were located in the posterior wall of the uterus and fundus. 

All the 17 surgeries were done in a laparoscopic manner and lasted between 60 and 120 minutes, with an average duration of 90 minutes. The incisions on the uterus were transverse and the haemostasis was assured with monopolar and bipolar instruments. Haemostatic clips were not used for the uterine arteries in any of the cases. After enucleating, the suture of the uterus was performed in two layers, for a better haemostasis and to prevent the uterine rupture in case of future pregnancies. At the end of the surgery, a drainage tube was placed in the recto-uterine pouch of all the patients, in order to be able to notice as soon as possible if there is any case of postoperative bleeding. 

All the 17 surgeries were made without incidents. Patients were hospitalized between 3 and 6 days. In addition, all of them were discharged with a good general condition, free of pains. 7 patients out of 14 became pregnant and gave birth.

## Conclusion

The epidemiological studies did not establish a relationship between leiomyomas and infertility but clinical studies showed that infertility is influenced by the presence of fibroids. There are more advantages with laparoscopic myomectomy than with laparotomy, even if the suturing of the uterus with laparoscopy is very demanding and requires a skilled endoscopic surgeon.

Laparoscopic myomectomy is one of the most challenging procedures in minimally invasive surgery.
